# Government innovation subsidy, executives’ academic capital and innovation quality: Evidence from pharmaceutical companies in China

**DOI:** 10.3389/fpsyg.2022.1092162

**Published:** 2023-01-11

**Authors:** Yuntian Xia, Min Fan, Xu Zuo, Wenjing Hao, Yiwen Jia

**Affiliations:** ^1^School of Economics and Management, Hefei Normal University, Hefei, China; ^2^School of Mechanical Engineering, Hefei University of Technology, Hefei, China; ^3^Department of Gastroenterology, Hefei First People’s Hospital, Hefei, China

**Keywords:** CEO’s academic capital, patents, quality, innovation, subsidy

## Abstract

The government employs innovation subsidies as a key incentive strategy to promote companies to innovate more technically. This study analyses how innovation subsidies influences the quality of corporate innovation. We create an innovation quality index for pharmaceutical corporations using categorizing data from patent applications submitted by pharmaceutical companies. Using data from 180 listed Chinese pharmaceutical companies between 2010 and 2020, this study proposes a panel regression model to assess the influence of government innovation subsidies on innovation quality, as well as the moderating effect of CEOs’ academic capital. How well innovations are subsidized is also affected by the heterogeneity of property rights. Innovation subsidy has a greater and more positive impact on non-SOEs. This article demonstrates that CEOs with academic credentials and executives with ties to the pharmaceutical industry have a variety of moderate effects. The research offers novel suggestions for enhancing business creativity and the innovation subsidy programme.

## 1. Introduction

In the post-COVID-19 era, the improvement of the quality of pharmaceutical technology innovation and the high quality development of the industry have an important role for social stability and economic development. Governments play a crucial role in nurturing and promoting the innovation of enterprises (Zhang and Nuttall, 2011; Aghmiuni et al., 2019). In order to advance goals of the public interest, government subsidies may establish policies to support innovations generally or specifically target a certain type of new technology (Shu et al., 2014; Huang et al., 2019). There are numerous types of assistance that government subsidies can provide, including tax refunds and innovation subsidy (Ren, 2022; Song and Zhao, 2022). The government faces a significant challenge in enhancing the quality of innovation.

China offers a variety of innovation incentives that cover a vast array of sectors. There are program-based innovation subsidy like the National High Technology Research and Development Program (863 Program) and the Torch Program. Innovation subsidy are available for eligible enterprises and their scientific and technological R&D projects through various programs (Gao et al., 2021). 70.8% of listed companies got government subsidies in 2007, with 40.5% percent receiving innovation subsidy. In 2019, the proportion of enterprises receiving government subsidies rose to 96.5%, with 1,717 listed companies receiving innovation subsidy.

Scholars have explored the positive impact of government subsidies on the inputs and outputs of enterprises’ innovation. Standing for the positive effect of government subsidies, the output of innovation has been investigated. Government subsidies can assist in compensating for market flaws in the innovation process, driving innovative inputs at the firm level, and fostering technical innovation activities among enterprises (Romano, 1989; Bérubé and Mohnen, 2009; Arqué Castells, 2013; Jaffe and Le, 2015; Bronzini and Piselli, 2016; Xia, 2022). The negative view of government subsidies holds that selected government subsidies inhibit the innovation of enterprises (Mamuneas and Ishaq, 1996; Shen and Lin, 2020; Huang et al., 2021). Rarely do articles examine the impact of government funding on the quality of innovation. When discussing the level of innovation, scholars have predominantly utilized the quantity of inventions s as a proxy for its quality (Ejermo, 2009; Taques et al., 2020). This type of indicator setting is unsuitable for measuring quality and may lead to the erroneous belief that enterprises pursue the quantity of inventions rather than only the quality of inventions (Pappas and Remer, 1985; Dang and Motohashi, 2015; Lazzarini et al., 2021). Consequently, new measurement metrics have emerged. The cited quantity indicator is subject to the influence of data withholding and indiscriminate citation (Carpenter et al., 1981; Harhoff et al., 2003; Arts et al., 2021; Higham et al., 2021) The legal dimension indicator represented by the grant rate and withdrawal rate rely solely on patent quality and is also affected by examination quality (Dang and Motohashi, 2015; Lin et al., 2021). The length of the payment period cannot appropriately reflect innovation’s social worth (Klepper, 1996; DiMasi et al., 2016). Is it therefore required to develop more accurate and rational measurement techniques?

Concerning the mechanism of innovation subsidy, the majority of research have focused on the direct impact path of innovation subsidy to boost enterprises’ R&D investment funds and hence stimulate R&D innovation (Almus and Czarnitzki, 2003; Bai et al., 2019; Hu et al., 2020). Several studies have also examined the behavioral additionality of innovation subsidy (Clarysse et al., 2009; Méndez-Morales and Muñoz, 2019). Using both theoretical (Kleer, 2010; Takalo and Tanayama, 2010; Su and Li, 2021) and empirical models (Feldman and Kelley, 2006; Meuleman and De Maeseneire, 2012; Li et al., 2021), they test the impact of innovation subsidy on the behavioral decisions of external investors. Through the examination and certification of firms’ R&D technological capabilities by government agencies, innovation subsidy can send signals that can attract more venture capital to support firms’ R&D innovation (Lerner, 2000; Liu et al., 2019; Li et al., 2021).

In this research, we focus on an important but understudied relationship between innovation quality and innovation subsidy. The majority of recognized R&D quality indicators, for example, are based on the number of inventions or citations, for which there is scant historical research. China’s patent application data, on the other hand, provides reasonable indicators for gauging the quality of R&D. Second, innovation grants are targeted awards with certain outcomes requirements. Could innovation subsidy enhance the quality of innovation in companies? We examine the effect on the pharmaceutical firms’ patenting quality of a government innovation subsidy. The listed pharmaceutical firms were used as a sample to examine the effect of government innovation subsidy on innovation quality, as well as the moderating effect of executives academic capital.

The followings are several contributions made by this article: Following the classification numbers of pharmaceutical companies’ patent applications, we conducted a comprehensive analysis using patent application information. Diversity and persistence are evaluated as characteristics of corporate innovation quality. This helps the Chinese government enhance business innovation quality in different perspectives. Second, we analyze the impact of government innovation subsidy on corporate innovation by manually collecting innovation subsidy from companies and removing the influence of non-innovation subsidy in order to make the study more relevant. Moreover, we evaluate the influence of innovation subsidy on the quality of innovation in Chinese pharmaceutical firms, giving micro-level evidence of the policy consequences of government innovation subsidy. Moreover, we use the academic capital of executives as a moderating variable to examine whether it help the innovation subsidy increase innovation quality.

The remaining sections are organized as follows: Following are discussions of both theoretical and empirical literature. Section 3 describes our dataset and its result factors. Section 4 describes the empirical methodology, key findings, and robustness test. Section 5 analyzes the results and their implications.

## 2. Literature review

### 2.1. Government innovation subsidy and innovation quality

Innovation is widely acknowledged as a key factor in corporate success and long-term economic progress. However, because to the problem of asymmetric information (Hall, 2005). and limited access to external financing (Rajan and Zingales, 2001). R&D efforts may be underfunded (Hong et al., 2016). The government helps firms boost their chances of success in R&D and supports their growth to promote innovative activities in businesses.

By granting innovation subsidy to firms, the government not only supplements the capital of the firms and decreases the risk associated with their R&D (Chang and Shih, 2004), but also distributes social resources to the enterprises and encourages them to engage in innovative activities (Zhao et al., 2022). Government innovation subsidy can increase the quality of firms’ innovation on two levels: by reducing the strain on their R&D budgets (Matallah, 2022) and by encouraging their R&D innovation (Fernández Sastre and Montalvo Quizhpi, 2019; Shah et al., 2022a).

On the one hand, firms can employ government innovation subsidy directly as innovation input funding (Lin and Luan, 2020). Subsidies can lessen the burden of endogenous finance necessary for innovation activities and minimize the investment costs of R&D and innovation for enterprises. Thus, enterprises have more capital available for R&D and innovation. The government, as a third-party subject, intervenes through financial measures such as innovation subsidy to pass information to external investors and so enhance innovation quality (Băzăvan, 2019). The government’s role is as follows: (1) Government innovation subsidy necessitates a thorough analysis and evaluation of the level of technological innovation capabilities of companies as well as the technical components, development possibilities, and economic contributions of grant-seeking initiatives. This policy contains a wealth of useful and essential market information (Zúñiga-Vicente et al., 2014; Wu and Zhao, 2022; Shah et al., 2022b). (2) The acquisition of an innovation subsidy by a corporation is an official acknowledgment of its R&D technology level, and the research project obtain innovation subsidy undoubtedly sends a positive signal regarding its technological superiority (Schneckenberg et al., 2015). External investors use a company’s eligibility for innovation subsidy as a beneficial information resource when making credit decisions, so effectively avoiding the potential adverse selection problem. Therefore, external investors may be more likely to invest in a business that has received an innovation award. (3) Innovation subsidy might be viewed as an invisible government credit guarantee to the enterprise (Zhu et al., 2012). As a primary focus of government assistance and attention, it can minimize the risk assessment of external investors (Hsu and Li, 2020) and cause them to have more consistent expectations regarding the reparability of credit funds, so increasing their trust and confidence in subsidized firms. In conclusion, innovation subsidy can assist enterprises ease financial strain and improve their innovation quality.

Second, government innovation subsidy can enhance the technological innovation quality of firms. Innovation subsidy can share the risk of R&D and innovation activities undertaken by enterprises (Yu et al., 2016). And the capture of government resources effects enterprises’ technological innovation strategies directly and enhances firms’ confidence in investing in cutting-edge technology. Subsidies from the government help enterprises define the direction of innovation at the policy level (Edler and Georghiou, 2007). Moreover, innovation subsidy facilitate sponsorship-based connections between enterprises and the government. These connections will also foster partnership-based connections between enterprises and other technological collaborators, such as universities and research institutions, as well as industry associations (Lee et al., 2001). Furthermore, government innovation subsidy can help enterprises in establishing partnerships with universities and research institutes (Hou et al., 2018), which can help enterprises expand their innovation expert knowledge (Ahn et al., 2020). Besides, the official transmission of information regarding technological advantages will result in the ongoing collection of social technological resources and joint R&D cooperation (Halili, 2020; Hattori et al., 2022). This will further promote the innovation capability enhancement of enterprises. The government takes the initiative in bringing enterprises together to collaborate, thereby enabling them to make breakthrough advances in highly specialized niches. Finally, after the enterprises have received innovation subsidy, the government will supervise and manage the implementation of their projects, regulating and directing them to engage in continuous R&D and innovation.

*H1*: Government innovation subsidy can promote the innovation quality of pharmaceutical enterprises.

### 2.2. The role of executives’ academic capital

According to the social resource hypothesis, the higher the social rank of the relator, the richer his or her social resources and the greater the helpful impact he or she generates (Lin, 1982; Ibarra and Andrews, 1993; Borgatti and Cross, 2003). In turn, a person’s social standing is typically expressed in terms of his or her professional influence and assessed by the status and prestige of their profession. Knowledge is a major factor in innovation (Savory, 2009; Mas et al., 2020). As a result, we want to find out if executives with academic capital can influence the relationship between innovation grants and firm innovation quality.

First, by appointing academic personnel in university or institution to the board of directors (Wolverton and Poch, 2000; Compagnucci and Spigarelli, 2020), enterprises can receive better internal support and a more streamlined implementation of innovation-related plans and actions due to their professional reputation and influence (Slaughter and Leslie, 1997; Zhou et al., 2022). Innovation in technology is long-term and high-risk. Enterprises are essentially rent-seekers (Fischer, 2007), acquiring funding for non-productive endeavors. This approach can be accompanied by significant market risk. In reaction to hostile takeovers, financial analysis tracking, and stock liquidity demands in the capital markets, corporate executives of listed enterprises prioritize short-term objectives (Sun et al., 2022) and reduce their investment in long-term innovation projects. Academic executives (Terpstra and Rozell, 1998), on the other hand, are more concentrated on technological innovation, have a profound understanding of innovation’s significance (Clements and Izan, 2008), and are more inclined to utilize innovation funds received by their companies in innovation activities (Finkelstein and Hambrick, 1990). Therefore, academic executives can, to some extent, restrain the short-sightedness of corporate executive teams (Ogbanufe et al., 2021) and encourage the use of innovation grants by enterprises (Darouichi et al., 2021) in order to improve their innovation efforts and ensure their long-term growth.

By virtue of their expert rights, CEOs with academic careers can effectively steer innovation during conversations with other corporate directors. And they can accelerate the senior management’s efforts to generate consensus (Xie et al., 2021). The bulk of very influential leaders are technology specialists. They can leverage their accumulated knowledge, R&D inertia, R&D skills, and R&D process expertise in a particular field to cut R&D time, lower R&D costs, and improve R&D efficiency for enterprises (Wang et al., 2021). Eventually, it will result in innovative outcomes of high quality. The government’s innovation grants are subject to a tighter evaluation of innovation outcomes and applicants must be academically-trained CEOs (Jin et al., 2022). Therefore, executives with professional backgrounds are more likely to get government innovation grants. Executives who apply for innovation grants have a higher grasp of technological innovation within firms and are prepared to adopt their ideas (Wang and Fung, 2022), which reduces moral hazard and improves the quality of corporate innovation. Finally, academic executives with access to strong university resources can facilitate the organization of partnerships between enterprises and research institutes or universities, as well as aid enterprises in achieving profound and revolutionary growth in a particular market area (Wang et al., 2018). Access to the interpersonal and social resources of universities and research institutes provided by academic executives gives firms an advantage when purchasing innovative resources such as personnel, technological equipment, and data. This will assist organizations in reducing the difficulty and cost of acquiring innovative factors and enhancing the innovation’s efficacy and quality (Shao et al., 2020). Consequently, the following conclusion can be drawn:

*H2*: Executives with academic career are able to improve the effect of government innovation subsidy on the innovation quality of pharmaceutical companies.

It has been shown that employees with extensive and rigorous training in the pharmaceutical industry make decisions based on their experience, evaluate situations with better composure, and provide more prudent and solid judgments (Babapour et al., 2018). On one, the process of studying biological, medicinal, and chemical disciplines is specialized and intricate. Executives who have had extensive professional training have acquired academic rigor and a sense of perseverance in the face of adversity, making them more rational and at ease while tackling problems and obstacles that arise during the innovation process. In addition, they have a unique understanding of the pharmaceutical industry’s cutting-edge research (Hung et al., 2005; Ye et al., 2022). In order to deliver superior results, executives with professional expertise can make better decisions and perform R&D more efficiently, regardless of the diversity or persistence of innovation. In addition, the critical thinking and independent thinking skills acquired through academic experience enable them to avoid following the crowed when making decisions and to insist on substantive innovation (Shen et al., 2020), which promotes the diversity of the executive team’s ideas (Kaiser et al., 2018) and contributes to the improvement of the executive team’s innovation quality.

Finally, according to the social capital theory, professionally affiliated executives may help organizations improve innovation quality. The executive professionals have gathered a broad network of undergraduate, graduate, and doctorate resources (Boni et al., 2009). Their social capital may represent an advantage of human interactions and technology that has not been multiplied (Jia et al., 2022). Through the bridge of academic executives, firms are able to quickly and precisely hire qualified technical R&D personnel (Cui, 2022). Therefore, the breadth and depth of information assists enterprises in improving the quality of innovation. Through the “bridge” of professional executives, firms can engage with universities and research institutes to share their technology and equipment resources and leverage on open innovation to boost the quality of innovation (Romanovich et al., 2014). By engaging in and monitoring the research, academic CEOs establish innovative collaborations that foster the sharing of information and provide enterprises with an early R&D and innovation advantage. Consequently, we get the following conclusion:

*H3*: Executives with professional ties can enhance the impact of government innovation subsidy on the innovation quality of pharmaceutical companies.

## 3. Research methodology

### 3.1. Sample and data

The data of Chinese A-share listed enterprises from 2010 to 2020 are matched with patent application data to conduct an empirical test of the above theoretical premise. For this paper, there are two main sources of data. First, the fundamental corporate and financial data are examined in the CSMAR databases. Companies that received ST or *ST treatment during the observation interval, companies that also issued B or H shares, and companies with a large amount of missing data are all excluded from this study. Second, information on patent applications used in this study came from the website of China’s State Intellectual Property Office.

### 3.2. Variables

#### 3.2.1. Dependent variables

The Dependent variable is **innovation quality**. We used two main indicators to verify our tests.

The first is **Diversity** using the patent breadth method Akcigit et al. (2016) and Aghion et al. (2019). It provides a novel perspective on the patent knowledge breadth technique, which assesses the degree of patent complexity (Kemeny et al., 2022). It depicts patent quality based on the complexity and depth of information included in patents, helping to overcome the limitations of applying simply the quantitative dimension of patents to measure company innovation. We determine the quality of patents based on the number of IPC classifications filed to the State Intellectual Property Office of China by enterprises. To evaluate the enterprise’s quality in the main tests, innovation patents and utility model patents are picked, while design patents are omitted. In robustness testing, Diversity1, including design patents, are utilized. The breadth of patent knowledge is weighted according to the logic of the Herfindahl–Hirschman index (HH) as it relates to the notion of gauging industrial concentration (Hall et al., 2001).


(1) $$PaKnowledge=1-\sum \alpha^{2}$$

Where α represents the percentage of each group inside the patent categorization number. As the PaKnowledge increases, the disparity between the various patent classification numbers becomes more pronounced. Consequently, the patent’s quality may increase in proportion to the company’s breadth of expertise utilized in its development. We estimate the innovation quality of business i in year t using Diversity. We utilize the PaKnowledge median as the Diversity to limit the influence of extreme values that are plainly unevenly distributed on the data.

The second is **Persistence.** This indicator is the technological continuity between patents filed in year t and the firm’s existing patent portfolio prior to year t − 1. This metric indicates whether a company stays inside or departs from an established research field. The method is followed by Jaffe (1986), Jaffe (1989), and Balsmeier et al. (2017). The measurement he used is known as the angular separation of the vectors and corresponds to the cosine of the angle between them.


(2) $$P_{ij}=\overset{k}{\underset{k=1}{\sum }}f_{ikt}f_{jkt-1}/{\left(\overset{k}{\underset{k=1}{\sum }}f_{ikt}^{2}\right)}^{1/2}{\left(\overset{k}{\underset{k=1}{\sum }}f_{jkt-1}^{2}\right)}^{1/2}$$

When K represents the IPC4 classification, and if a cited patent has two different IPC classification codes, each classification is counted as a separate patent. $f_{ikt}$ represents the proportion of patents belonging to category k for listed firm i in application year t. and $f_{ikt-1}$ represents the proportion of all patents for listed business I in application year *t* − 1 that belong within category k. The greater the $P_{ij}$, the greater the similarity between the company’s patent portfolio in year *t* and year *t* − 1. That would suggest that pharmaceutical enterprises are more persistent in their pursuit of innovation. Innovation patents and utility model patents are chosen to evaluate the enterprise’s quality in the primary tests, whereas design patents are omitted. Diversity1, including design patents, are utilized in robustness testing.

#### 3.2.2. Independent variable

The independent variable is **Innovation subsidy (GIS)**, which aim to reduce the financial strains of pharmaceutical companies engaged in technical advancement. Only the total amount of government subsidies is disclosed by the listed companies. In this work, we employ the “keyword search” technique to locate specific items in the government subsidy documentation. The results of a keyword search are condensed. First, search terms related to science and technology, such as “research and development,” “development,” “innovation,” “science and technology,” “technology development,” “technology project grant,” “important technology application,” etc., are employed. Second, keywords such as The terms “Star and Fire Plan,” “Torch Plan,” “863,” “Small Giant,” “high-tech enterprise,” “productivity promotion center,” “gazelle enterprise,” “incubator,” “First Set,” “Science and Technology Support Program,” “Standardization Strategy,” and “Golden Sun” are researched. Then, decisions are made regarding new products, patents, copyrights, and intellectual property rights. In addition, we searched for key terms associated with innovative talents and technical cooperation, such as “attracting talents and wisdom,” “talent storage,” “doctoral laboratory,” “elite plan,” “giant plan,” “University-Industry Research,” “University-Enterprise Cooperation,” “Overseas Team,” “Overseas Engineers,” and “foreign cooperation,” etc. As filters, research is conducted on novel cancer therapies, spores, antibiotics, and other forms of biological medical technology. Finally, we calculate the annual total innovation subsidies awarded to each listed company.

#### 3.2.3. Moderating variable

The moderating variable is **Academic capital of executives**. This paper adheres to the definition of corporate executives by Bamber et al. (2010) and Dyreng et al. (2010), which refers to the senior management personnel who are directly involved in the business decision-making of the enterprise, including the chairman, chief executive officer, general manager, executive general manager, deputy general manager, executive vice general manager, chief accountant, and financial officer, as well as the members of the board of directors and executive committee. Executives’ academic backgrounds mirror their work backgrounds. In addition to being the total of social and human capital, it can assist executives in utilizing available resources. Academic executives have better innovation skills and more innovation resources than general executives. They can assist corporate innovation with more research and are more likely to see innovative results. According to the requirements of the empirical analysis, the key independent variables are organized as follows in this study. Two moderating factors that affect executives’ academic backgrounds include their graduating major (GGP) and whether they have worked in academic or research institutions (GGA).

In order to determine if an executive’s profession is relevant to the pharmaceutical sector, we filters the annual report data to determine their educational history. The specialization of a pharmaceutical firm executive is noted as relevant if his or her major is related to medicine, pharmacy, biology, or chemistry. Finally, before computing the natural logarithm, the annual number of executives with a professional background in each listed company is aggregated (GGP). The GGA method is similar to the GGP method. We calculate the natural logarithm of the number of executives of listed firms who work in universities or research institutes each year using data obtained from annual reports.

#### 3.2.4. Control variables

In order to control as much as possible for each contributing element of firm innovation quality and to prevent endogeneity difficulties caused by neglecting essential factors. The control variables include ROE (return of equity), RevenueG (growth of revenue), Lev (ratio of total debt to total assets), TATO (turnout of total assests), Days (Cash operating cycle) Finally, a year dummy (Year) is introduced in the regression model(Czarnitzki and Hussinger, 2004; Kaul, 2011; So, 2022).

### 3.3. Models


(3) $$Innovation_{i,t}=\beta_{0}+\beta_{1}GIS_{i,t-1}+\beta Control_{i,t-1}+\alpha_{i}+\alpha_{t}+\varepsilon_{i,t}$$

where $Innovation_{i,t}\;$is the quality of company innovation, $GIS_{i,t-1}$ is the level of government innovation subsidy with a one-period lag, $Control_{i,t-1}$ is a set of firm-level control variables with a one-period lag presented in this study, $\alpha_{i}$ is an individual fixed effect, $\alpha_{t}$ is a period fixed effect, and $\varepsilon_{i,t}$ is a random error term.


(4) $$\begin{array}{c} Innovation_{i,t}=\beta_{0}+\beta_{1}GIS_{i,t-1}+\beta_{2}Acedemic_{i,t-1}\\ +\beta_{3}GIS_{i,t-1}\times Acedemic_{i,t-1}+\beta Control_{i,t-1}\\ +\alpha_{i}+\alpha_{t}+\varepsilon_{i,t} \end{array}$$

Equation (4) displays the empirical model used to investigate the moderating influence of government innovation subsidy. $Acedemic_{i,t}$ is indicators of executives’ academic capital. The interaction term between the amount of government innovation subsidy and the academic is denoted by $GIS_{i,t}\times Acedemic_{i,t}$.

## 4. Empirical results

### 4.1. Descriptive statistics and correlations

Table 1 provides descriptive statistics for all important factors. Diversity has a mean value of 0.312 and a standard deviation of 0.348. The mean value of Persistence is 0.352, with a standard deviation of 0.417, and the maximum value is 1, indicating that some of the companies consistently focus on one field. These numbers indicate that knowledge of a particular patent varies significantly. The mean for the natural logarithm GIS is 2.081and the standard deviation GSI is 4.683. The distributions of other variables are identical to those reported in previous studies. As a result of the fact that all VIFs are below the 10-point threshold (Kutner et al., 2004; Peter, 2008), there are no evident linkages between variables. In addition, a Hausman test indicates that a fixed effect model should be utilized in this research. The majority of indices have been evaluated in accordance with prior research, and only a few significant control variables have been added to our models (Zheng et al., 2022).

**Table 1:** Descriptive statistics.

Variable	*N*	Mean	SD	Median	Min	Max	VIF
Diversity	1,484	0.312	0.348	0	0	0.902	
Persistence	1,484	0.352	0.417	0	0	1	
GSI	1,484	2.081	4.683	0	0	16.41	1.01
ROE	1,484	−0.114	3.342	−0.0100	−22.80	13.98	1.05
RevenueG	1,484	0.222	0.562	0.102	−0.514	4.191	1.18
Lev	1,484	0.368	0.210	0.343	0.0330	0.927	1.19
TATO	1,484	0.623	0.378	0.530	0.126	2.223	1.30
Days	1,484	309.0	445.1	201.7	45.65	3,600	1.28

### 4.2. Main regression analysis

Table 2 presents the results of government innovation subsidy and innovation quality.

**Table 2:** Empirical results of the impact of GIS on innovation quality.

	(1)	(2)	(3)	(4)	(5)	(6)
Variables	Diversity	Diversity	Diversity	Persistence	Persistence	Persistence
GSI	0.0049^***^(0.0016)	0.0042^***^(0.0016)	0.0042^***^(0.002)	0.0044^***^(0.0021)	0.0043^***^(0.0021)	0.0043^***^(0.0022)
ROE		0.0844^***^(0.0295)	0.0668^**^(0.0302)		0.0900^**^(0.0388)	0.0695^*^(0.0397)
RevenueG		0.0358^***^(0.0130)	0.0358^***^(0.0120)		0.0880^**^(0.0381)	0.0700^*^(0.0390)
Lev		0.0756^**^(0.0297)	0.0756^**^(0.0334)		0.126^***^(0.0380)	0.109^***^(0.0387)
TATO		−0.0778^**^(0.0368)	−0.0778^*^(0.0406)		−0.104^**^(0.0469)	−0.104^**^(0.0471)
Days		0.109^***^(0.0293)	0.109^***^(0.0343)		0.0913^**^(0.0379)	0.0764^**^(0.0386)
StockTO		0.0598^**^(0.0296)	0.0598^*^(0.0341)		0.00665^***^(0.00198)	0.00665^***^(0.00243)
Constant	0.235^***^(0.0232)	0.277^***^(0.0395)	0.277^***^(0.0449)	0.343^***^(0.00854)	0.363^***^(0.0414)	0.363^***^(0.0373)
Control	NO	YES	YES	NO	YES	YES
Year fixed	NO	YES	YES	NO	YES	YES
Individual fixed	NO	NO	YES	NO	NO	YES
R-squared	0.252	0.244	0.244	0.204	0.118	0.118
Number of stkcd	180	180	180	180	180	180

Standard errors in parentheses.

*** *p* < 0.01.

** *p* < 0.05.

* *p* < 0.1.

Column (1–3) shows the regression diversity of innovation for model (1). The GIS coefficient in column (1) is 0.0049, which is statistically significant at the 0.01 level. Column (2–3) displays the findings following the addition of several control factors, suggesting that government innovation subsidy remains significant despite considering the endogenous difficulties produced by the missing variables. Subsidies for innovation can have a favorable impact on the diversity of innovations. There may be further incentives for enterprises to broaden their knowledge boundary. The estimation results of innovation persistence without and with control variables are displayed in columns (4), (5), and (6), respectively. The 0.0044 value of the GIS primary coefficients is statistically significant at the 0.01 level. This research indicates that government innovation subsidy can increase the innovation persistence of enterprises. Therefore, these results support H1.

### 4.3. Heterogeneity of property rights

The development of advanced technology is the result of advancing in one area or innovating across numerous fields. Innovation is essential for enterprises to increase their viability. There are significant differences between state-owned enterprises (SOEs) and non-state-owned enterprises (non-SOEs) in terms of resource acquisition, management privileges, and internal governance structure, which are attributable to their property rights in China (Lin et al., 2010; Choi et al., 2011). Enterprises’ uneven property rights have a significant influence on their technological innovation practices, aspirations, and operating conditions (Aghion et al., 2013).

SOEs and non-SOEs coexist in China’s mixed market. Due to their close ties to the local government, SOEs are more likely to get government subsidies than non-SOEs (Wu, 2017; Xu et al., 2020). SOEs in China are less concerned with survival and more concerned with compliance (Yang and Yao, 2011). However, non-SOEs are more worried about survival. In various pressure situations, attitudes regarding government innovation subsidy varies. Non-SOEs are under higher pressure to survive, and as a result, they must prioritize and maximize their R&D outcomes’ conversion rate and quality (Zhou et al., 2016). They think that innovation subsidy will facilitate the development of superior technological advances. As a result, we divide the sample into two subsamples (SOE and non-SOEs) and re-estimate all models. 5.The results can be find in Table 3. Diversity and persistence metrics for SOEs are insignificant. In contrast, diversity and persistence, two indices of non-SOEs, are significantly positive correlated. Government innovation subsidy have a strong positive effect on the innovation quality non-SOEs, as demonstrated by this evidence.

**Table 3:** Empirical results of heterogeneity of property rights.

Variables	SOE		N_SOE	
	Diversity	Persistence	Diversity	Persistence
GSI	0.00191(0.0033)	0.00256(0.0037)	0.00431^***^(0.0008)	0.00475^***^(0.0004)
ROE	−0.0102^*^(0.0055)	−0.0184^***^(0.0062)	0.0601^*^(0.0361)	0.106^**^(0.0479)
RevenueG	0.0610^**^(0.0255)	0.0381(0.0292)	0.0341^**^(0.0156)	0.106^**^(0.0479)
Lev	0.0878^**^(0.0353)	−0.0756(0.261)	−0.0422(0.0583)	0.100^**^(0.0471)
TATO	−0.160(0.122)	−0.203(0.138)	−0.0473(0.0410)	−0.0948*(0.0530)
Days	0.113^**^(0.0466)	0.121^***^(0.0349)	0.0878^**^(0.0353)	0.100^**^(0.0471)
StockTO	−0.0080(0.0117)	−0.0087(0.0131)	0.0035^**^(0.00156)	0.0068^***^(0.0021)
Constant	0.370^***^(0.1411)	0.522^***^(0.1573)	0.255^***^(0.0440)	0.368^***^(0.0444)
Year fixed	YES	YES	YES	YES
Individual fixed	YES	YES	YES	YES
R-squared	0.119	0.176	0.146	0.118
Number of stkcd	33	33	147	147

Standard errors in parentheses.

*** *p* < 0.01.

** *p* < 0.05.

* *p* < 0.1.

### 4.4. Robustness tests

#### 4.4.1. Replacing independent variables and dependent variables

This study will conduct robustness tests in the following areas to further assess the dependability of the results of the initial regression (Boeing, 2016). Diversity1 and Persistence1 are substitutions for Diversity and Persistence, respectively, the results can be find in column (1–2) in Table 4. Moreover we replace the GSIration. The outcomes are essentially consistent with the standard regression.

**Table 4:** Robustness tests of replacing independent variables and dependent variables.

Variables	(1)Diversity1	(2)Persistence1	(3)Diversity1	(4)Persistence1
GSI	0.0087^**^(0.0036)	0.0102^***^(0.003)		
GSIratio			10.65^***^(0.1567)	10.07^***^(0.1539)
ROE	0.145^**^(0.074)	0.161^**^(0.0677)	−0.0018(0.0025)	−0.0023(0.0018)
RevenueG	0.134^*^(0.0724)	0.0949^***^(0.0027)	0.0265(0.0171)	0.0345^***^(0.0131)
Lev	−0.439^***^(0.107)	−0.361^***^(0.0919)	0.0033(0.0709)	−0.0087(0.0542)
TATO	−0.0616(0.0671)	−0.110^*^(0.0567)	−0.101^**^(0.0469)	−0.106^***^(0.0359)
Days	0.1121^***^(0.0089)	0.1313^***^(0.0078)	0.1141^***^(0.0093)	0.1138^***^(0.0038)
StockTO	0.0046^*^(0.0028)	0.0093^*^(0.0023)	0.0067^***^(0.0020)	0.0040^***^(0.0015)
Constant	0.704^***^(0.084)	0.712^***^(0.0723)	0.365^***^(0.0414)	0.352^***^(0.0074)
Year fixed	YES	YES	YES	YES
Individual fixed	YES	YES	YES	YES
R-squared	0.189	0.213	0.217	0.217
Number of stkcd	180	180	180	180

Standard errors in parentheses.

*** *p* < 0.01.

** *p* < 0.05.

* *p* < 0.1.

#### 4.4.2. Instrumental variable method

We conducted an instrumental variable analysis to alleviate the endogeneity concern caused by missing variables. We employed the L.GSI, which is calculated as a one-period lag of government innovation subsidy, as the instrumental variables (IV) in a two-stage least squares model. The results of the first stage of regression are shown in column (1) of Table 5. The estimated coefficient of the instrumental variable (IV) is 0.5461, which is statistically significant at the 1% level, and the F-statistic is significantly more than 10, showing that there is no problem with a weak instrumental variable. The outcomes of the second step of regression are displayed in column (2) of Table 5, where the estimated coefficients of diversity and persistence are 0.0092 and 0.0080, respectively, which are statistically significant at the 1% level. The results continue to indicate that government innovation subsidy can greatly boost the innovation quality of pharmaceutical enterprises, confirming the robustness of the results of the previous regression analysis.

**Table 5:** Robustness tests of instrumental variable method.

Variables	IV = L.GSI		
	Diversity	Diversity	Persistence
IV	0.5461^***^(0.0341)		
GSI		0.0092^***^(0.0012)	0.0080^***^(0.0022)
ROE	0.6681^***^(0.0314)	0.0798^*^(0.0422)	0.882^**^(0.0512)
RevenueG	−0.0649(0.01646)	0.0965^**^(0.0423)	0.0667(0.0505)
Lev	0.2238(0.5101)	−0.239^***^(0.0467)	−0.418^***^(0.0536)
TATO	0.5148^*^(0.3107)	−0.103^***^(0.0230)	−0.0890^***^(0.0272)
Days	0.0002^***^(0.0000)	−0.0001^***^(0.0000)	−0.0002^***^(0.0000)
StockTO	0.001780.0161	−0.0041^***^(0.0010)	−0.0039^***^(0.0015)
Constant	0.235^***^(0.0232)	0.454^***^(0.0391)	0.561^***^(0.0483)
Control	NO	YES	YES
Year fixed	NO	YES	YES
Individual fixed	NO	NO	YES
R-squared	0.192	0.242	0.264
Number of stkcd	180	180	180

Standard errors in parentheses.

*** *p* < 0.01.

** *p* < 0.05.

* *p* < 0.1.

### 4.5. Moderating effect test

Our evidence so far implies that government innovation subsidy can effectively improve innovation quality. Table 6 displays the empirical estimation findings for testing the moderating influence of executives’ academic capital. In column (1), the coefficient of the key interaction term GIS*GGA is positive and significant. This result suggests that the effect of e government innovation subsidy on diversity of innovation quality is more pronounced in enterprises with more executives working in university or institution. However, the results in column (3) is not significant. That may indicates that executives with academic career cannot moderate the relationship between government innovation subsidy and persistence of innovation quality. This finding partly supports H2. In column (3) and (4), GIS*GGP is a variable with significantly positive coefficients in diversity and persistence of innovation quality. Thus, executives with professional ties can enhance the impact of government innovation subsidy on the innovation quality of pharmaceutical companies. Thus, H3 is supported.

**Table 6:** Empirical results of executives’ academic experience as moderator.

Variables	Moderator_GGA		Moderator_GGP	
	(1)	(2)	(3)	(4)
	Diversity	Persistence	Diversity	Persistence
GIS	0.0431^***^(0.00158)	0.0372^***^(0.00208)	0.0442^***^(0.00159)	0.0371^***^(0.00209)
GGA	0.000628(0.00450)	0.0296(0.0239)		
GIS*GGA	0.0360^**^(0.0181)	0.00409(0.00592)		
GGP			0.00589(0.00592)	0.0260(0.0236)
GIS*GGP			0.0388^**^(0.0180)	0.0145^***^(0.00778)
ROE	−0.00121(0.00188)	−0.00109(0.00247)	−0.00132(0.00188)	−0.00114(0.00247)
RevenueG	0.0364^***^(0.0130)	0.0275(0.0172)	0.0367^***^(0.0130)	0.0277(0.0171)
Assestdebit	−0.0163(0.0545)	0.00430(0.0717)	−0.00968(0.0549)	0.00719(0.0721)
TAssestTurnout	−0.0773^**^(0.0368)	−0.0916^*^(0.0483)	−0.0783^**^(0.0367)	−0.0924^*^(0.0483)
Days	9.89e-06(3.15e-05)	1.48e-05(4.14e-05)	1.44e-05(3.15e-05)	1.81e-05(4.14e-05)
StockTO	0.00344^**^(0.00151)	0.00627^***^(0.00199)	0.00342^**^(0.00151)	0.00627^***^(0.00198)
Constant	0.231^***^	0.265^***^	0.243^***^	0.280^***^
	(0.0457)	(0.0601)	(0.0419)	(0.0551)
R-squared	0.247	0.231	0.350	0.334
Number of stkcd	180	180	180	180

Standard errors in parentheses.

*** *p* < 0.01.

** *p* < 0.05.

* *p* < 0.1.

## 5. Conclusion and policy implications

### 5.1. Conclusion

This study investigates the impact of government innovation subsidy on three facets of innovation quality. We find that the benefits of innovation subsidy boosting innovation quality, by studying the classifications of patents’ applications. Furthermore, we evaluate the variability of property rights. In addition, the research investigates the moderating effect of academic capital among executives. Several key conclusions are as follows:

1. The government innovation subsidy has a significant effect on the diversity and persistence of innovations. The outcomes suggest that the pharmaceutical industry’s innovation subsidy policies are effectively applied. Dang and Motohashi (2015) suggested that patent subsidy programs increase patent counts more than 30%.For China’s new energy vehicle industry, Sun et al. (2019) demonstrated a positive association between innovation subsidies and innovation performance. Lin et al. (2021) discovered that positive effect of the innovation subsidy policies on patent quality, by using the forward citations, patent claims, and number of inventors as quality indicators.
2. Taking property rights into account, we find that the innovation quality of non-SOEs is more sensitive to government innovation subsidy, and that the innovation quality of enterprises improves as the proportion of institutional investors increases. Innovation subsidy have had an insignificant effect on the innovation quality of SOEs. Government innovation subsidy can alleviate the funding pressure on non-SOEs and help them improve, allowing enterprises to innovate more effectively.
3. The academic capital of executives has the moderate effect on government innovation subsidy and pharmaceutical innovation quality. Executives with professional connections can boost the impact of government innovation subsidy on the innovation quality of pharmaceutical companies. The influence of a government innovation subsidy on the diversity of invention quality is more pronounced in enterprises with a higher share of university or institution-trained chief executive officers.

### 5.2. Policy implications

On the basis of continuing to promote the growth of innovation quantity, the quality of innovation will be used as a significant criterion for the distribution of innovation subsidy, and the assessment results of enterprise innovation quality will be incorporated into the subsequent stage of subsidy allocation decisions. First, government should establish an evaluation and assessment index system for invention quality. The index system for measuring and assessing the quality of innovations should be developed from the perspectives of originality and influence. Moreover, the qualification examination for subsidy beneficiaries should place greater emphasis on the quality of innovation. While focusing on the quantity of patents, particularly invention patents, the quality of the patents of sponsored companies should be improved.

Moreover, there should be a distinction between SOEs and non-SOEs in terms of the incentives. The organization and distribution of subsidies for innovation projects or scientific research projects for non-SOEs can be changed, and the amount of post-grant funding can be appropriately increased. Subsidies will also be awarded based on the quality of the innovation results, particularly the subsequent impact and application. The main goal for SOEs is to boost their passion and willingness to innovate, and the amount of R&D funding and other relevant incentives should be strongly correlated with the volume and caliber of innovation produced by SOEs.

This study also reveals that executives’ academic capital has a favorable impact on government innovation subsidy. The government should consider more thorough evaluation indices, such as the standard of project R&D manuals and the technical R&D background of management teams, in the selection of high-quality support targets that reflect the R&D innovation capability of enterprises and the logic and viability of their R&D projects. The corporate innovation team should have a stronger impact on R&D and innovation competence to optimize the incentive effect of the innovation subsidy. We should also provide the company’s innovation team more influence over the distribution of human, financial, and material resources as well as the choice of technology routes in order to improve the policy impact of innovation subsidy.

### 5.3. Managerial implications

What we find is also interesting and valuable for managers. The findings have managerial implications for executives choices. As the executives’ background of academic and professions can promote the quality of innovations. Therefore, CEOs with academic and professional backgrounds might be a priority for companies that conduct R&D operations and provide R&D outputs. This is due to the fact that they can contribute more to the innovation quality in terms of both diversity and persistence.

### 5.4. Limitation and future research

Due to limited data availability, the study’s sample size is rather small. In this study, only the pharmaceutical industry was chosen as the sample for research, and quantitative data on the classification of its patent applications, statistics on innovation outputs, and executive academic information were collected. Future investigation will determine the applicability of this study’s findings to other fields. Second, the government innovation subsidy selection project is influenced by the policy department’s decision rather than the project’s own estimation of future market and technology development trends. Does this hamper the creativity of companies? This subject is beyond the scope of this paper and deserves more investigation.

## Data availability statement

The datasets presented in this study can be found in online repositories. The names of the repository/repositories and accession number(s) can be found at: https://www.gtarsc.com/.

## Author contributions

YX: conceptualization and methodology. YJ: funding acquisition and supervision. MF: data analysis. XZ: data curation and writing. WH: writing and proofreading. All authors contributed to the article and approved the submitted version.

## Funding

This work was supported by Anhui Provincial Department of Education (2021jyxm1247, 2021jxtd235, KJ2021A0088, SK2021ZD0045, and SK2021A0507, 2022AH040286) and Anhui Medical University (2022xkj105).

## Conflict of interest

The authors declare that the research was conducted in the absence of any commercial or financial relationships that could be construed as a potential conflict of interest.

## Publisher’s note

All claims expressed in this article are solely those of the authors and do not necessarily represent those of their affiliated organizations, or those of the publisher, the editors and the reviewers. Any product that may be evaluated in this article, or claim that may be made by its manufacturer, is not guaranteed or endorsed by the publisher.
